# Coping strategies among nurses in South-west Ethiopia: descriptive, institution-based cross-sectional study

**DOI:** 10.1186/s13104-018-3557-5

**Published:** 2018-07-03

**Authors:** Tadesse Dagget Tesfaye

**Affiliations:** Department of Adult Health Nursing, College of Medicine and Health Sciences, Bahir Dar University, Bahir Dar, Ethiopia

**Keywords:** Coping strategies, Nurses, Jimma Zone public hospitals

## Abstract

**Objective:**

This study aimed to describe coping strategies for job stress among nurses working in Jimma Zone public hospitals, South-west Ethiopia. The study conducted from March to April 2014 through census using English version structured self-administered questionnaire.

**Result:**

This study indicated percentage mean overall score of 65.07% for adaptive coping approach and 56.86% for a maladaptive approach. Nurses mostly used coping strategy were; just concentrating on what they have to do, make a plan of action and following it, developing coworker/peer support, and having a close friend to tell. While, coping strategy that least used among nurses were; do not want to come to work when stressed, directly expressing anger on family or friends, trying to feel better by taking drinks like tea, coffee, soft drinks more than usual and accept the situation because there is nothing to do. In summary, an adaptive approach was dominant style; social support and plan-full problem solving were the most preferred strategies. While escape-avoidance coping strategy least used. Further researches need to be conducted to explore its predictors.

**Electronic supplementary material:**

The online version of this article (10.1186/s13104-018-3557-5) contains supplementary material, which is available to authorized users.

## Introduction

Coping has been described as any cognitive and/or behavioral efforts to manage, minimize, or tolerate events that individuals perceive as potentially threatening to their well-being. It does not imply success in dealing with situations, the responses to stressors can also be maladaptive. According to Folkman and Lazarus transactional theories of stress, it places emphasis on subjective perceptions of stressors, and individual differences in ways of coping, viewing problems, past experience, personality-type, etc [1, 2]. All these may be important in informing and affecting the workplace–individual stress interaction [3]. The two forms of coping are problem-focused and emotion-focused coping strategies. Problem-focused coping strategies are problem-solving tactics. These strategies encompass efforts to define the problem, generate alternative solutions, weigh the costs and benefits of various actions, take actions to change what is modifiable, and, if necessary, learn new skills. Emotion-focused coping strategies are directed toward decreasing emotional distress. These tactics include efforts like distancing, avoiding, selective attention, blaming, minimizing, wishful thinking, venting emotions, seeking social support, exercising, and meditating. Emotion-focused coping is the more common form of coping used when events are not change able [4].

A study in Malaysia in public hospital indicated six most preferred coping mechanisms by nurses to reduce job stress were; to have a close friend to confine in, compartmentalize work and home life, hobbies, leisure activities, recreation and turn to prayer or spiritual thoughts, plan instead of responding to pressure, and building work-group norms of cooperation, not competition. While, use of tranquilizers/drugs, leave a job for another and take it out on family/friends were coping mechanisms never preferred by nurses in descending order. In summary by theme, nurses coping mechanisms in a descending order were control, social support, escape, and symptom management [5].

Studies have shown that majority of nurses prefer more active coping strategies/positive reappraisal, plan-full problem solving and seeking social support and a few use avoidance coping strategies like blame someone else for their problem, sleep more than usual and eat more [6–8].

The work setting is the main reason for job stress [9]. Job stress is one of the major psychosocial risks at work and a great concern for many organizations and employees [10]. Little is known about the coping strategies among nurses in our country Ethiopia especially in nurses working in Jimma Zone public hospitals.

This research has identified coping mechanism implemented by nurses to reduce job stress in Jimma zone public hospitals.

Several investigations on the coping mechanism for job stress were done in many developed countries and some African countries. However, research on how to cope job stress among nurses working in hospitals in Ethiopia to the best knowledge of the investigator it is still insufficient.

The aim of this study was to assess coping strategy among nurses working in Jimma Zone public hospitals, South-west Ethiopia.

## Main text

### Methods and materials

#### Study area and period

This study conducted in Jimma Zone, Oromia National Regional State, where its zonal city is Jimma that far 325 km from Addis Ababa [11]. Three public hospitals (Jimma University specialized hospital (JUSH), Shenen Gibe and Limu Genet hospital) involved in the study. The study was carried out from March to April 2014 in these three public hospitals.

#### Study design

A cross-sectional study was conducted among nurses with greater or equal to 6 months of work experience and those who were available at work at Jimma Zone public hospitals during data collection period.

#### Sample size

A total of 433 nurses were available in the study hospitals, among those only 73 were less than 6 months of experience and the rest 360 involved in the study by a census.

#### Data collection instrument

English version structured self-administered questionnaire developed through adaption from expanded nursing stress scale used for data collection [12]. It contains three sections that suit the purpose of the study.

Part-I consists of socio-demographic questions. Part-II contains eighteen items on the coping mechanism used to measure coping with job stress. The items are divided into five types of coping mechanisms (5 sub-scales). They are plan-full problem solving (has 5 items), self-control mechanism (has 2 items), social support (has 3 items), symptom management mechanism (has 3 items), and escape/avoidance mechanism (has 5 items). Respondents requested to score each item rated on a four-point Likert item with a score ranging from 1 to 4 where ‘1’ “I never do this”, ‘2’ “I sometimes do this”, ‘3’ “I frequently do this” and ‘4’ “I always do this”. To identify coping strategy, the total score for adaptive approach obtained by adding all the scores of 13 items and their sum score range a minimum score of 13 and maximum of 52 which indicates the higher the score the preferred coping strategy used by the nurse while for maladaptive approach the score of the 5 items added and their sum score range a minimum score of 5 and maximum of 20 which indicates the higher the score the preferred coping strategy used by the nurse. The questionnaire was pretested at Woliso St. Luke Hospital before the start of actual data collection and necessary comments and feedback taken and incorporated. Overall Cronbach’s alpha score for overall adaptive coping measuring items 0.86, maladaptive coping measuring items 0.76.

#### Data collection

Data was collection was facilitated by a five diploma nurses after training. Collected data was reviewed and checked for completeness by the facilitator/data collectors and principal investigator.

#### Data processing and analysis

The data was edited, entered into Epi-Data version 3.1 and exported to IBM SPSS Statistics Version 20 for analysis. The results were summarized and presented in tables, and charts. Percentage, frequency and mean calculated.

To identify coping strategy: the total score for adaptive approach obtained by adding all the scores of 13 items and their sum score range a minimum score of 13 and maximum of 52 which indicates the higher the score the preferred coping strategy used by the nurse. While for the maladaptive approach the score of the 5 items added and their sum score range a minimum score of 5 and maximum of 20 which indicates the higher the score the preferred coping strategy used by the nurse.

For coping strategies subscales, the participant’s responses on each item of respective subscale score summed to give subscale score. Subscales mean score converted to percentage mean score. It indicates the higher the score the more the nurse preferred the way of coping strategy.

### Operational definitions

#### Coping strategy

A strategy that helps people to reduce stress and solve problems which are measured through adaptive approach and maladaptive approach subscales. Percentage means score calculated (actual computed mean score divide by maximum potential score multiplied by hundred) for each subscale to weight their rank.

- *Adaptive approach* attempts to manage or alter the problem causing the stress measured through plan-full problem solving, symptom management, social support and self controlling and these subscales having a total of 13 items and their sum score range a minimum score of 13 and maximum of 52 which indicates the higher the score the more preferred coping strategy used by the nurse.
- *Maladaptive approach* attempts to regulate stressful situation through escape-avoidance indicators which are: make plans to change/leave the career, avoid stressful situation, take it out on family or friends, accept the situation and to absentee from work and their sum score range a minimum score of 5 and maximum of 20 which indicates the higher the score the more preferred coping strategy used by the nurse [13].

### Results

#### Socio-demographic characteristics

**Fig. 1:**
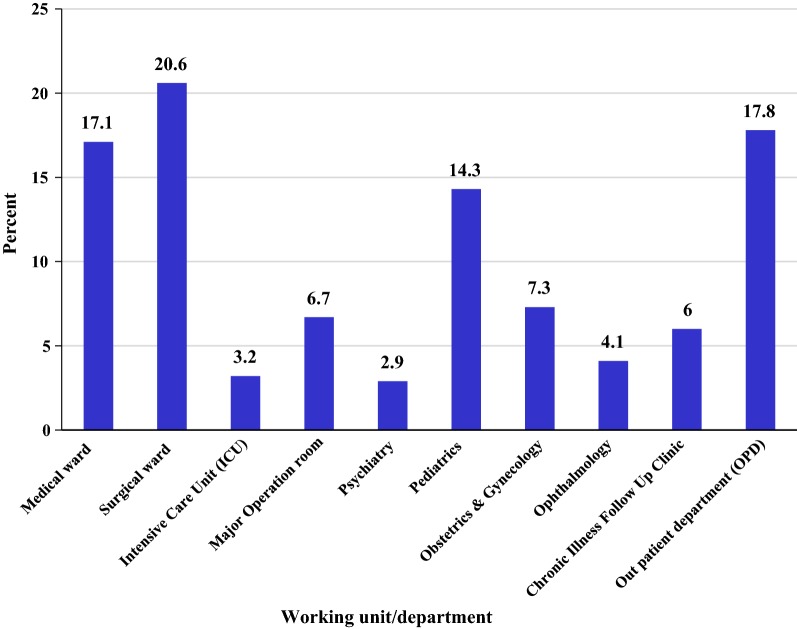
Working unit/department among nurses working in Jimma Zone public hospitals

Three hundred forty-one nurses who were working in JUSH, Shenen-Gibe and Limu Genet Hospital were given a self-administered questionnaire, 315 responded (92.38%). The majority (85.7%) were from JUSH, 6.6% from Shenen Gibe Hospital and the rest 7.9% from Limu Genet Hospital. Half of the study participants (50.8%) were males. More than half (54.6%) were single followed by 43.2% (136) married and 2.2% (7) divorced. More than three-fourths (77.5%) had served less than 5 years. Regarding participants position in the hospital; staff nurses 89.8% (283), head nurse 8.6% (27), supervisor nurse 0.6% (2) and matron nurses 1% (3). Participants were between 21 and 58 years old (mean age 27.95 ± 6.83 years). Almost 9 among 10 (89.9%) were diploma nurses while the rest are B.Sc. Majority 20.6% (65) of nurses were from surgical ward (Fig. 1).

#### Description of coping strategies

Nurses working in Jimma Zone public hospital mostly used coping strategy were; just concentrating on what they have to do next (2.99 ± 0.861), make a plan of action and following it (2.9 ± 0.917), developing coworker/peer support (2.76 ± 0.955), and having a close friend to tell (2.66 ± 1.038).

Coping strategy that least used among nurses were; do not want to come to work when stressed (1.93 ± 0.99), directly expressing anger on family or friends (1.95 ± 0.963), trying to feel better by taking drinks like tea, coffee, soft drinks more than usual (2.23 ± 1.046) and accept the situation because there is nothing to do (2.24 ± 1.051) (Table 1).

**Table 1:** Frequency distribution of each item of coping strategy among nurses working in Jimma Zone public hospitals, South-west Ethiopia (N = 315)

Subscale	Item	I never do this		I sometimes do this		I frequently do this		I always do this		Mean	Std. dev
		N	%	N	%	N	%	N	%		
Plan-full problem solving	I provide independent time for workplace and home life activity	58	18.4	88	27.9	90	28.6	79	25.1	2.60	1.05
	I build a satisfactory relationship with supervisor/matron	69	21.9	106	33.7	89	28.3	51	16.2	2.39	1.00
	I seek help from the head nurse/supervisor or physicians	57	18.1	136	43.2	86	27.3	36	11.4	2.32	0.90
	I just concentrated on what I have to do next	13	4.1	79	25.1	121	38.4	102	32.4	2.99	0.86
	I made a plan of action and followed it	21	6.7	86	27.3	111	35.2	97	30.8	2.90	0.92
Self-control mechanism	I tried to keep my feelings to myself	39	12.4	113	35.9	102	32.4	61	19.4	2.59	0.94
	I tried to keep my feelings to myself from interfering with another thing too much	30	9.5	127	40.3	106	33.7	52	16.5	2.57	0.88
Social support	I have a close friend to share/tell	50	15.9	90	28.6	91	28.9	84	26.7	2.66	1.04
	I discuss a problem with family members	49	15.6	97	30.8	86	27.3	83	26.3	2.64	1.04
	I develop coworker/peer support	34	10.8	89	28.3	112	35.6	80	25.4	2.76	0.96
Symptom management	I tried to make myself feel better by taking drinks like tea, coffee, soft drinks more than usual	97	30.8	96	30.5	75	23.8	47	14.9	2.23	1.05
	I tried to make myself feel better by engaging in hobbies, leisure activities, and recreation	55	17.5	101	32.1	96	30.5	63	20.0	2.53	1.00
	I turn to prayer or spiritual thoughts	49	15.6	95	30.2	88	27.9	83	26.3	2.65	1.03
Escape/avoidance	I make plans to change/leave/the career	56	17.8	87	27.6	99	31.4	73	23.2	2.60	1.03
	I did not want to come to work when I am stressed	131	41.6	108	34.3	42	13.3	34	10.8	1.93	0.99
	I avoid being in stressor situation if I can	51	16.2	88	27.9	96	30.5	80	25.4	2.65	1.03
	I directly express my anger on my family or friends	124	39.4	113	35.9	48	15.2	30	9.5	1.95	0.96
	I accept this situation because there is nothing I can do to change it	92	29.2	109	34.6	61	19.4	53	16.8	2.24	1.05

Coping strategy had five subscales from these the most used strategy were social support (67.12%) followed by plan-full problem solving (66.02%) while escape-avoidance (56.86%) was the least used strategy to manage stress among nurses (Table 2).

**Table 2:** Percentage mean score of coping strategy subscales and overall coping approaches among nurses working in Jimma Zone public hospitals, South-west Ethiopia (n = 315)

Coping strategy	Sub-scale	Mean	Std. deviation	Mean %	Minimum %	Maximum %
Adaptive	Social support	8.06	2.27	67.12	25	100
	Plan-full problem solving	13.2	2.82	66.02	25	100
	Self-control	5.16	1.53	64.45	25	100
	Symptom management	7.41	2.34	61.75	25	100
	Total adaptive approach score	33.83	6.73	65.07	25	100
Maladaptive	Escape-avoidance	11.37	3.28	56.86	25	100

### Discussion

This study respondents (n = 315) indicated percentage mean overall score for adaptive coping approach and maladaptive approach were 65.07 and 56.86%, respectively.

The highest rated coping strategy items that nurses use always and frequently were just concentrating on what they have to do next. While seeking help from head nurses/supervisors or physicians, do not want to come to work when stressed and directly expressing anger towards family or friends were the least used coping mechanisms.

Regarding overall coping strategy used among nurses, this study indicated that adaptive coping strategy used mostly than maladaptive coping (escape-avoidance). This is congruent with a study done in Mangalore study that stated the majority of the nurses used active coping strategies (adaptive coping) and a few use avoidance coping strategies [6] and supported by study conducted in Ibadan showed that stress coping responses of nurses were largely based on planning and active coping [7].

From adaptive coping strategies, social support followed by plan-full problem solving were dominantly used coping strategies among nurses. This is congruent with a study done in Mangalore and Gaza-Palestine that stated nurses’ commonly used coping mechanisms include problem-solving and social support [6, 13].

Despite the fact that majority of study participants prefer the adaptive approach, in this study still there were nurses who were using maladaptive approach. Studies suggest using problem-focused/adaptive approach coping strategies than maladaptive approach/escape-avoidance [14]. Hence, strategies have to be designed so as to encourage nurses to familiarize the adaptive approach.

In conclusion, nurses were using adaptive approach; social support and plan-full problem solving were the most preferred stress management mechanisms while escape-avoidance coping strategy least used.

Further studies need to be conducted to explore a lot and intervene on coping strategies for job-related stress including private/non-governmental hospitals and health centers.

## Limitations

This study has not assessed associated factors for coping strategies for job stress.

## Additional file

**Additional file 1.** Coping strategy dataset. This is the dataset analyzed for the manuscript.

## Abbreviations

JUSH: Jimma University specialized hospital
St.: Saint
Std. Dev: standard deviation
